# Impact on touch DNA of an alcohol-based hand sanitizer used in COVID-19 prevention

**DOI:** 10.1007/s00414-023-02979-2

**Published:** 2023-02-24

**Authors:** Carla Bini, Arianna Giorgetti, Giulia Fazio, Sara Amurri, Guido Pelletti, Susi Pelotti

**Affiliations:** Section of Legal Medicine, Department of Medical and Surgical Sciences, University of Bologna, Via Irnerio, 49, 40126 Bologna, Italy

**Keywords:** Touch DNA, Hand sanitizer, COVID-19, Forensic analysis, STR DNA profiling

## Abstract

In the last years, forensic research has been focused on touch DNA in order to improve its evidential value in criminal activity investigations as well as to understand the variables impacting touch DNA. One of the emerging variables is represented by the use of alcohol-based sanitizers, which was suggested for hand hygiene during the COVID-19 pandemic. The aims of the present study were to assess the effect of a hand sanitizer on touch DNA deposition, transfer, and recovery and also to evaluate STR typing success, quality of DNA profiles, and personal identification. Before and after the use of an alcohol-based hand sanitizer, 20 volunteers deposited on glass surfaces 120 fingerprints, containing skin-derived or salivary DNA. Samples were quantified by real-time quantitative PCR (q-PCR), and 76 samples yielding > 15 pg/μl were typed for 21 autosomal STRs by GlobalFiler® PCR Amplification Kit. DNA profiles were classified into single source, mixed, and inconclusive profiles, and a LR assessment was performed by comparison to the reference samples using LRmix Studio software. After the use of hand sanitizer, samples yielded lower quantities of recovered transferred DNA, especially considering samples containing salivary DNA (*p* < 0.05 by Friedman test). All the 76 amplified samples (63.3% of the total) showed at least 10 typed loci, and 83–100% of profiles were consistent with the reference ones on the basis of a LR value ≥ 10^6^. Results showed that, although the hand sanitizer reduces the DNA recovering, touch DNA samples might still be useful for forensic personal identification even when hand sanitizers are used.

**Supplementary Information:**

The online version contains supplementary material available at 10.1007/s00414-023-02979-2.

## Introduction

The improvement of the forensic DNA analysis techniques and technologies has opened up the opportunity to generate short-tandem repeat (STR) profiles from touched surfaces and items, which have been increasingly used in casework laboratories and courtrooms as sources of forensic DNA evidence. The transfer of human DNA by handling objects or touching surfaces was firstly described by van Oorschot and Jones, who also highlighted that the amount of DNA deposited by hands, the so-called touch DNA, can be person-dependent [1]*.*

Since then, several studies have investigated a person’s propensity to deposit DNA — the shedder status — most concurring with the early findings that some individuals consistently deposit comparatively more or less DNA than others and are commonly referred to as “good” and “poor” shedders [2–4]*.* An individual’s behavior, e.g., by frequent handwashing or using gloves, can also affect the shedder status, especially when considering activities that involve the transfer of biological materials characterized by a relatively high DNA content, such as blood or saliva [5]*.* When saliva is transferred on hands, e.g., by licking the thumbs to turn pages or by biting the fingernails, saliva-derived DNA might be transferred together with skin-derived one. In this type of DNA transfer, due to the high content of genetic material, the shedder status may be less relevant with regard to touch DNA samples [6].

Touch DNA samples might fall below the recommended thresholds at any stage of the analysis, and amplifications might be affected by stochastic effects such as allele and locus dropout, peak imbalance, and drop-in [7]. Optimized methodologies to improve DNA collection, extraction, amplification, and typing were suggested [8]. The amplification and typing success of STR loci can be improved by removing amplification inhibitors, increasing the number of cycles, redesigning the primer sequences, or altering the master mix components, using chemical adjuvants, or purifying the post-amplification PCR products [9]. However, forensic laboratories have been reluctant to implement these methodologies due to validation, contamination, and artifacts issues [9].

Understanding the variables affecting DNA transfer, persistence, prevalence, and recovery (DNA-TPPR), but also the STR amplification and typing success, has become increasingly relevant in criminal activity investigations to provide insight into how a person’s DNA got to where it is collected [7].

Although some factors appear to steadily influence the shedder status of a person over time, other conditional factors such as environment and activity also have an impact in a given situation [10].

The global pandemic that we are still going through has completely changed our daily habits and our lifestyle: we are invited and urged to sanitize our hands, to wash them as many times as possible, and to assume behaviors exclusively aimed at reducing the transmission of the SARS-CoV-2 virus [11]. Alcohol-based sanitizers have been recommended by the World Health Organization (WHO) and Centers for Disease Control and Prevention (CDC) as an effective measure to prevent microbial disease transmission [11] and several scientific studies on their efficacy and safety with different formulations have been published [11, 12]. Active components are usually represented by ethanol, isopropyl alcohol, and n-propanol, with a minimum concentration recommended for activity against SARS-CoV-2 virus of 60–75% v/v [10, 13]. Hand sanitizers might contain excipients like thickening agents, humectants that moisten the skin preventing its dryness but make the formulation sticky, fragrances, and colorants, depending on the formulation [14]. Gels and foams are the most commonly available products and differ in the ability to interact with the skin, contact time, and handling of the products [14]. Hand sanitizers are intended for quick application on the hands, with no need for water or soap, and their effectiveness also depends on the application technique [13]. Indeed, to ensure the killing of germs, it is recommended to rub the hands together for at least 20 s until they are dry [15].

The extensive and unprecedent use of hand sanitizers to combat the COVID-19 pandemic with alcohol-based solutions (formula) has never been previously experienced in modern times, and no study has yet focused on the effects that sanitizers used in the prevention of COVID-19 infection can have on touch DNA.

Nevertheless, the alcoholic component could interfere with the DNA yield [16], and the sticky excipients could alter the collection and retaining of skin-derived DNA. Moreover, the action of rubbing the hands might spread self- and non-self-DNA across the skin surface, modifying the amount of DNA that could be transferred.

The purpose of the present research was to evaluate whether the use of alcohol-based hand sanitizers could affect the deposition, transfer, and the recovery of self- and non-self-touch DNA. In addition, given that saliva may be a more prevalent source of genetic material during transfer events than the epithelial cells deposited from a hand [6], the effect of hand sanitizer was evaluated on touch deposits from fingertips previously moistened with saliva. Furthermore, the present study aimed to evaluate the STR typing success, the quality, and the source of DNA profiles obtained from touch DNA after using alcohol-based hand sanitizers.

## Materials and methods

### Experimental design

The study was conducted in compliance with ethical standards and was approved by the Bioethical Committee of the University of Bologna (Prot. n. 283,821 approved on November 4th, 2021). Twenty volunteers, 10 women and 10 men, aged in the range 25–50 years, were recruited. Volunteers who agreed to participate in the research project were asked to fill out and sign the informed consent.

The hand sanitizer used in this study (*Bactygel hand gel sanitizer*’ from the Kemica group) consists of ethyl denatured alcohol (63.3%), water, hydroxypropyl methylcellulose, and C.I. 42,090 (ACID BLUE 9). It appears viscous, liquid, and light blue with a density of 0.894 g/ml.

### DNA fingerprint deposition

Microscope glass slides (50 mm × 75 mm) were used as substrates for fingermark deposition. To degrade any possible extraneous DNA, previous to the experiment, each microscope slide was cleaned with 3% bleach solution, rinsed with bi-distilled water and absolute ethanol, and, before use, irradiated under ultraviolet (UV) light overnight in order to ensure no contaminating DNA was present. The contact with the glass surface was made by the volunteer by pressing palm down the three fingers of each hand for 15 s, exerting pressure but without rubbing.

**Fig. 1:**
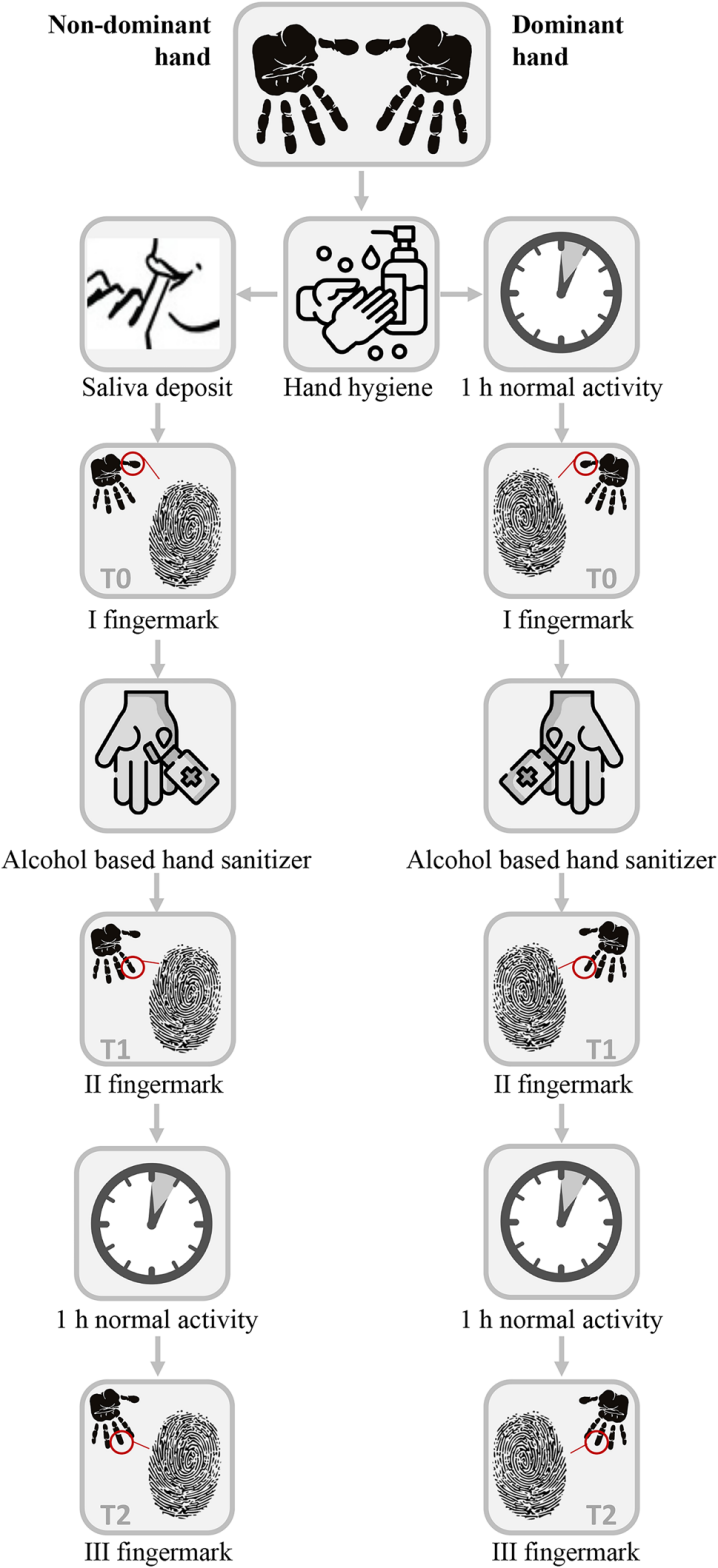
Schematic diagram of the experimental design regarding the process and timing of DNA fingerprint deposition

Touch DNA deposits from the dominant and non-dominant hands were collected according to the following protocol, as represented in Fig. 1. Volunteers were asked to wash their hands with hand soap and water and to dry them in air. During the time interval between handwashing and fingerprint deposition, they were asked to refrain from washing or sanitizing their hands with alcohol, otherwise proceeding with their daily routine.

#### *Dominant hand (touch DNA)*

Within an hour from handwashing, the first touch DNA sample was obtained by placing the first finger of the dominant hand on a microscope slide (T0). Participants were then asked to use the alcohol-based detergent on both hands as per normal cleansing, to distribute it on the hands by rubbing and to let it air-dry for 30 s. A second DNA touch sample was obtained by fingerprint deposition of the second finger on a new slide (T1). After 1 h, during which volunteers were again asked to refrain from washing or sanitizing hands, a third and final touch DNA sample was obtained by fingerprint deposition of the third finger (T2).

#### Non-dominant hand (salivary DNA added)

Immediately after hand washing, volunteers were asked to moisten the first three fingertips of their non-dominant hand with saliva. Within the following hour, the first deposit of biological material was obtained by the first finger of the non-dominant hand. Following the same protocol of the dominant hand, the second and third DNA touch deposits were obtained.

In total, 120 samples were collected, 6 for each volunteer, of which 3 from the non-dominant and 3 from the dominant hand.

### Sampling procedure

Within 30 min from the deposition, the sampling of fingerprints was carried out from each slide using a flocked nylon swab 4N6FLOQSwabs™ Crime Scene (Copan Italia S.p.A., Italy). The swab was lightly moistened in RNase-/DNase-free water to rehydrate the cells on the surface and facilitate the recovery of cellular material. Negative background controls, i.e., slides that had not been touched, and slides on which the hand sanitizer was dropped, were swabbed and analyzed for the presence of background DNA.

From volunteers, the buccal swabs were collected by a sterile dry cotton swab (Copan Italia S.p.A., Italy) as reference samples. After DNA collection, the swabs were stored at − 20 °C until further processing.

### DNA extraction

Two different protocols were used for DNA extraction based on the amount of starting DNA.

DNA from samples collected on the slides was isolated using a commercial silica-based DNA extraction system by QIAmp DNA Investigator Kit (Qiagen, Hilden, Germany) following the manufacturer’s protocol for surface and buccal swabs, with a final elution volume of 30 μL. Buccal swabs were submitted to Chelex extraction method [17], using the ReadyAMP™ Genomic DNA Purification System Kit (Promega). For each extraction session, a negative control was used.

### DNA quantification and DNA profiling

DNA quantification was performed using the Quantifiler™ Trio DNA Quantification Kit on the QuantStudio 5 Real-Time PCR System for Human Identification (Applied Biosystems), following the manufacturer’s protocol. DNA samples with quantification values equal to or greater than 0.015 ng/μl in T0 and T1 or in T0 and T2 were chosen to be amplified by multiplex PCR. This choice was made in order to be able to compare the profiles obtained before and after the use of the alcohol-based hand sanitizer.

Seventy-six out of 120 samples were amplified using the GlobalFiler® PCR Amplification Kit (Thermo Fisher Scientific) in accordance with the manufacturer’s recommendations with the standard 29 cycles on the Veriti™ 96-Well Thermal Cycler (Applied Biosystems) instrument. Amplified products were separated and detected on the SeqStudio™ Genetic Analyzer (Applied Biosystems). Data collection and fragments analysis was conducted using GeneMapper® ID-X v 1.6 (Thermo Fisher Scientific) with an analytical threshold set to 100 relative fluorescence units (RFU).

### Data interpretation

The generated DNA profiles were classified into single source or mixed profiles, showing more than two allelic signals at two or more loci, or inconclusive profiles with less than 10 typed loci not suitable for comparison [18, 19]. The maximum allele count (MAC) and minimum number of contributors (MNC) to the profile were calculated. To complete the profile outcomes, mixed profiles were then classified as mixed profiles with a major contributor, when one or two alleles at each locus were in a ratio of peak height ≥ 3:1 relative to the other alleles of the same locus. On the contrary, when the allele peak height ratio was < 3:1, profiles were classified as mixed with no major contributor [19].

DNA profiles were compared to the reference samples of the donors, counting loci and alleles dropout (absence of an allele or a locus in the profile that is present in the reference DNA profile). The biostatistical evaluation for the LR assessment was performed using LRmix Studio software v. 2.1.5 [20], after estimating the dropout probability.

Single source profiles and mixed profiles providing a value of LR ≥ 10^6^ were in the present study deemed informative profiles to identify the donors. The threshold of LR ≥ 10^6^ was chosen because it provides an extremely strong support for the prosecution hypothesis (Hp) rather than the alternative defense hypothesis (Hd) [21].

### Statistical analysis

To check for a normal distribution, Anderson–Darling, D’Agostino and Pearson, Shapiro–Wilk, and Kolmogorov–Smirnov tests were run [22]. Since *p* was always < 0.05, showing a non-Gaussian distribution, descriptive statistics of the quantification data was performed in order to describe median and interquartile range (IQ) of the whole data, as well as for the male and female subsamples and for the dominant and non-dominant hand in the different sampling times (T0, T1 and T2). A comparison of DNA content was made between the male and the female population, for each hand and sampling time, by non-parametric *t* test (Mann–Whitney test). Similarly, the DNA content of the dominant hand and the non-dominant hand was compared for each sampling time by Wilcoxon test, non-parametric test for paired samples.

The comparison of the DNA content among the different touch deposits (T0, T1, T2), was made by Friedman test for paired non-parametric samples. When a statistically significant difference was shown by the Friedman test, a post hoc multiple comparison test was additionally carried out by Dunn’s multiple comparisons test, a non-parametric test which compared T0 with T1, T0 with T2, and T1 with T2. Chi-square analysis was performed to evaluate the genotyping results, testing for the association between different touch deposits (T0, T1, T2) and generated profiles, as well as between touch deposits and LR results, whether ≥ or < 10^6^.

In all statistical analyses, the significance level was set at < 0.05. Statistical analysis and graphs were obtained using Stata/MP 15.1 and GraphPad Prism version 8.2.1.

## Results

**Fig. 2:**
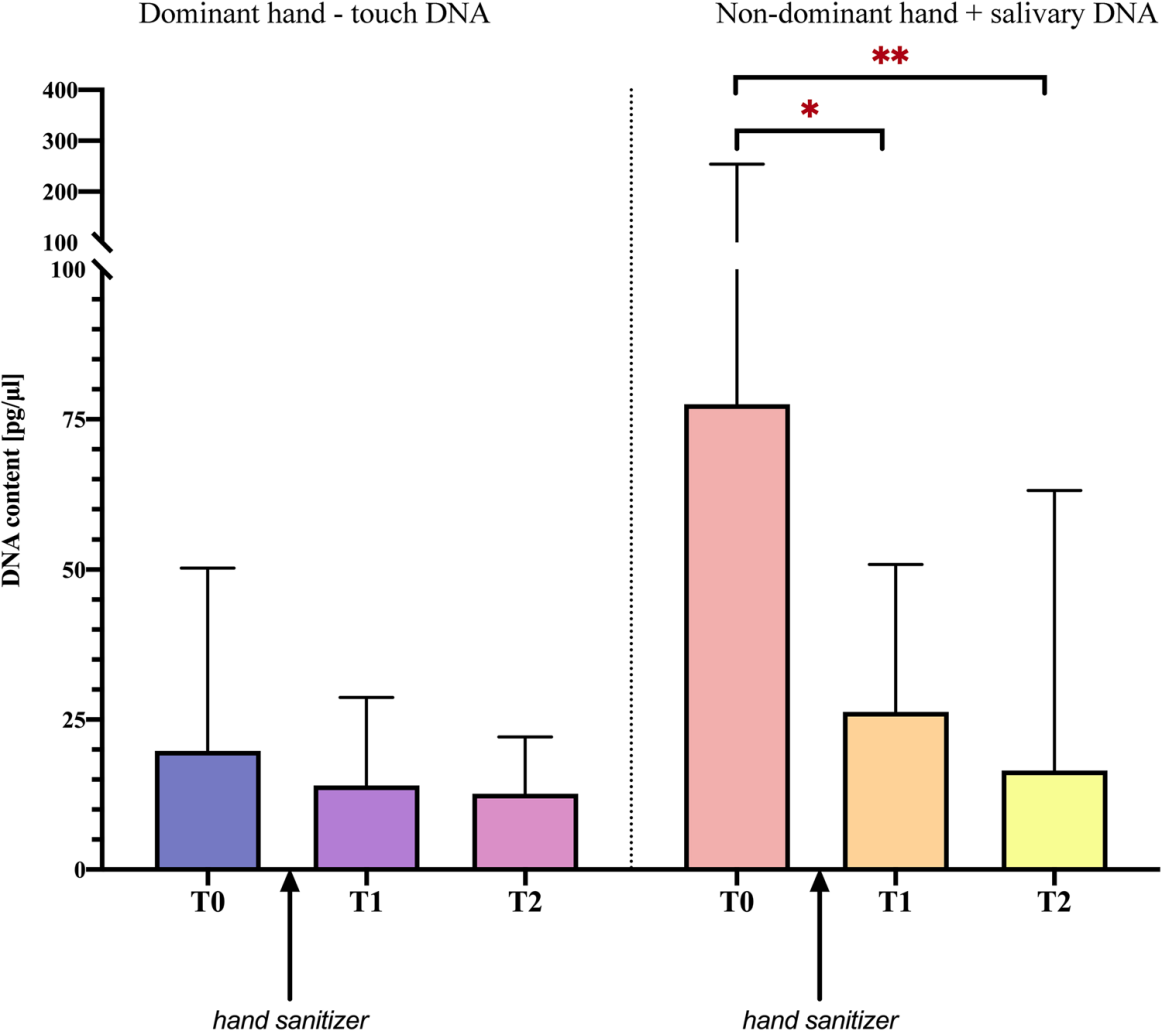
Comparison of T0, T1, and T2 for the dominant hand (left side of the figure) and non-dominant hand (right side of the figure). T0, before alcohol-based hand sanitizer; T1, immediately after the use of the alcohol-based hand sanitizer; T2, 1 h after the use of alcohol-based hand sanitizer. *,* p* = 0.0006; **, *p* = 0.0011

The results of the DNA quantification before the use of hand sanitizer (T0) immediately after the use of hand sanitizer (T1) and 1 h after the use of hand sanitizer (T2) are represented graphically in Fig. 2. Median values and IQ ranges for each timing are shown in Table 1. Overall, values ranged from 0 to 469 pg/μl at the dominant hand and from 0 pg/μl to 2.73 ng/μl at the non-dominant hand. Background control samples did not show quantifiable DNA (data not shown).

**Table 1:** Summary of the quantification and genotyping results for the dominant and the non-dominant hand

	T	Quant. pg/μl	N. samples amplified	Single source profiles	Mixed profiles		Informative profiles
					Without major contributor	With major contributor	
*Dominant hand*	T0	19.8 (6.4–50.3)	12	5 (41.7%)	6 (50%)	1 (8.3%)	10 (83.3%)
	T1	14.0 (5.0–28.7)	11	6 (54.5%)	1 (9.1%)	4 (36.4%)	11 (100%)
	T2	12.7 (5.5–22.1)	9	6 (66.7%)	3 (33.3%)	0 (0%)	8 (88.9%)
*Non-dominant hand*	T0	77.5 (20.1–254.0)	17	14 (82.4%)	1 (5.9%)	2 (11.8%)	16 (94.1%)
	T1	26.3 (8.1–50.1)	13	6 (46.2%)	5 (38.5%)	2 (15.4%)	13 (100%)
	T2	16.5 (9.8–63.1)	14	9 (64.3%)	4 (28.6%)	1 (7.1%)	14 (100%)

Quantification results (Quant.) are given as the median and the interquartile range by brackets. *T*, timing; *T0*, before alcohol-based hand sanitizer; *T1*, immediately after the use of the alcohol-based hand sanitizer; *T2*, 1 h after the use of alcohol-based hand sanitizer. *N*, number of samples from each hand and timing which were amplified. Informative profiles: on the basis of the LR ≥ 10^6^

Quantifiler™ Trio DNA Quantification Kit demonstrated that no inhibitors were present in all tested samples for the subsequent PCR amplification step and provided a degradation index ranging from index 3 to 8.3 in 4 out of 120 samples (3.3%), of which 2 at T0, one at T1, and one at T2.

The difference between the male and female population in the amount of human DNA deposited on glass was not significant at any sampling time and considering both hands (*p* > 0.05) (see more detailed in the Fig. A, Supplementary material).

By comparing the amount of DNA deposited with the dominant or the non-dominant hand, the difference was statistically significant at T0 (*p* = 0.024) and at T1 (*p* = 0.015) but not at T2 (*p* > 0.05) (Fig. B, Supplementary material).

By assessing the effect of the hand hygiene with alcohol-based hand sanitizer on the DNA content, the difference between the touch deposits obtained from the dominant hand across the three sampling times (T0, T1, and T2) was non statistically significant (*p* = 0.58). On the contrary, when considering samples added with salivary DNA, a statistically significant difference (*p* < 0.001) was shown. The difference was still significant after removing the subject with the higher value, who resulted in 2.73 ng/μl DNA at T0 and could be considered as an outlier with respect to the interquartile ranges (median 77.5 ng/μl, IQ range 20.1–254.0 ng/μl as shown in Table 1). The post hoc Dunn’s multiple comparison test, applied on samples from the non-dominant hand, particularly demonstrated a statistically significant difference between touch deposits before (T0) and immediately after the use of the hand sanitizer (T1) (*p* < 0.001). Moreover, a statistically significant difference was found between T0 and the deposition 1 h after the use of the hand sanitizer (T2) (*p* < 0.001), while T1 did not differ significantly from T2 (*p* > 0.5) (Fig. 2).

From the 120 total samples, 76 samples (63.3%) were above the threshold value of 0.015 ng/μl for the DNA profiling and were then selected and carried out for the amplification.

### Genotyping results

From the selected 76 samples, 29 were collected at T0 (72.5% of T0 samples), 24 at T1 (60% of T1 samples), and 23 at T2 (57.5% of T2 samples). Following the DNA profile classification reported above, no profile was deemed inconclusive. Overall, from the typing analysis, 46 STR profiles were classified as single source profiles (60.5%) and 30 (39.5%) as mixed profiles, of which 20 (26.3% of the total profiles) without a major contributor and 10 (13.2% of the total profiles) with a major contributor. Mixed profiles showed a maximum number of alleles/locus ≤ 5. In Table 1, the number of samples amplified from each hand and timing and the resultant DNA profiles are reported.

Considering the profile outcomes in the dominant hand, mixed profiles with no major contributor were the most common outcome at T0 (50%), followed by single source profiles (41.7%). At T1, mixed profiles with no major contributor decreased to 9.1%, while those with major contributor increased from 8.3 to 36.4%. At T2, single source profiles prevailed on the mixed ones (66.7%), and no mixed profiles with a major contributor were generated.

Profile outcomes in the non-dominant hand showed a prevalence of single source profiles across all timing and especially at T0 (82.4%). At T1, single source profiles decreased to 46.2%, and mixed profiles with no major contributor increased from 5.9 to 38.5%. At T2, the distribution of generated profiles was similar to T1.

**Fig. 3:**
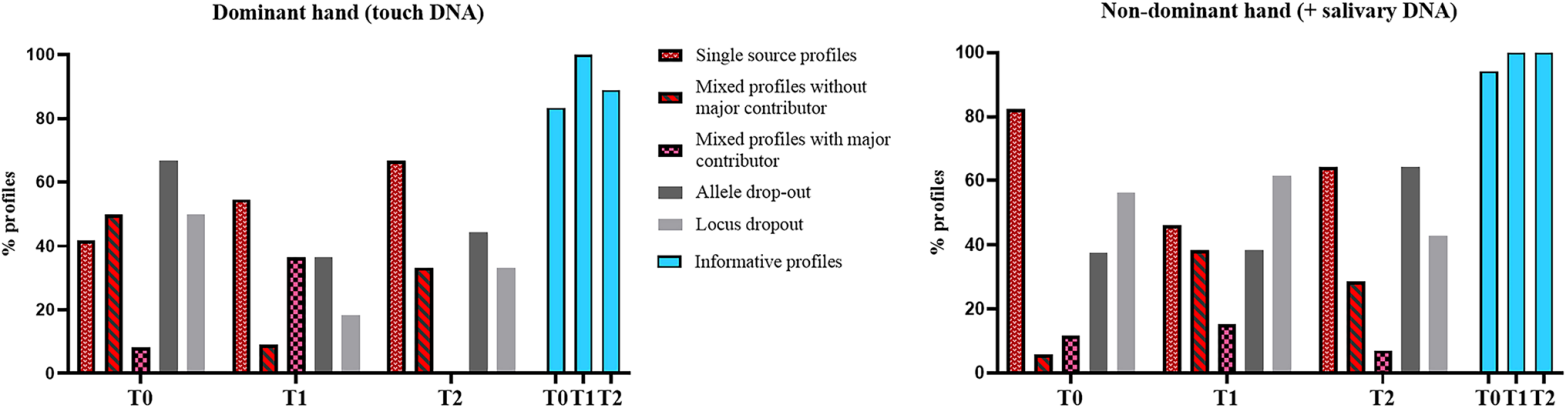
Genotyping results obtained from the dominant and non-dominant hand, shown as percentages on the total number of samples amplified. T0, before alcohol-based hand sanitizer; T1, immediately after the use of the alcohol-based hand sanitizer; T2, 1 h after the use of alcohol-based hand sanitizer

Generated STR profiles for the dominant and non-dominant hand across the three touch deposits are displayed in Fig. 3.

Samples showing allele dropout were 66.7% at T0, 36.4% at T1, and 44.4% at T2 in the dominant hand. Locus dropout was seen in 50.0% of samples at T0, 18.2% at T1, and 33.3% at T2 of the same hand. Considering the non-dominant hand, samples showing allele dropout were 37.5% at T0, 38.5% at T1, and 64.3% at T2. Locus dropout was seen in 56.3% of samples at T0, 61.5% at T1, and 42.9% at T2 of the same hand. Percentages of allele and locus dropout are shown in Fig. 3.

The results of the biostatistical calculation obtained with the LRmix Studio v. 2.1.5 software showed for the dominant hand a LR range of 10^−12^ < LR < 10^28^ and for the non-dominant hand a range of 10^2^ < LR < 10^32^. Samples showing an LR value < 10^6^ were 4 out of 76 (5.3%), of which 3 from the dominant hand (2 at T0, 1 at T2) and one from the non-dominant hand (at T0). Percentages of informative profiles (matching the donor profile with LR ≥ 10^6^) ranged from 83.3 to 100% and are reported, together with the number of amplified samples and profile composition, in Table 1.

Informative profiles are also graphically shown in Fig. 3.

No association was found between timing of touch deposits (T0, T1, T2) and STR profile outcome (*p* > 0.05) and between the timing of touch deposits (T0, T1, T2) and the LR values, whether ≥ or < 10^6^ (*p* > 0.05).

Detailed genotyping results for each sample, including generated STR profiles, MAC, MNC, the dropout count and LR values, are shown in the Supplementary material Table B.

## Discussion

The purpose of the research was to evaluate whether the hand sanitizers, which in COVID-19 pandemic began to be used daily, can affect the DNA recovery and the STR typing from samples containing skin-derived or also salivary DNA, a biological fluid with a high DNA quantity.

A precise hand washing and detergent use protocol was applied so that each volunteer deposited three dominant hand and three non-dominant hand fingerprints at three different deposition times on UV-treated glass surfaces.

We used a gel that is sticky in nature, of proven effective against bacteria and viruses, and widely distributed in local public offices and laboratories.

The amount of touch DNA recovered after fingerprint pressure on the glass surfaces with the dominant hand, especially before the use of the hand sanitizers, showed a high inter-individual variability (as shown by IQ ranges in Table 1), in the ranges reported by previous studies involving the same surface, similar length, and nature of contact [23–26]. This might be due to the shedding propensity among volunteers (although this was not tested in our study), to the different activities performed and surfaces touched during the 1 h of normal activity before the touch deposition [26]. The efficacy of touch DNA recovery can also be influenced by collection devices and surfaces [27].

The wide interquartile range found among the recruited volunteers and the various activities might explain why a significant difference between the DNA content released by female and male volunteers was not found, in apparent contrast with the increased DNA shedding propensity of male donors reported by other authors [23, 25]. However, it has been also reported that adult subjects might change status, from good to poor shedder, from time to time, with a low predictability [28, 29]. Given the absence of significant differences in the amount of DNA deposited by males and females, the two subpopulations were combined in our study to analyze the effect of the hand sanitizer on the recovery of touch DNA.

As expected [6], the amount of human DNA yielded after contact with the dominant hand was significantly lower compared to the one deposited by fingertips previously moistened with saliva. Nevertheless, the present work did not aim to assess the amount of salivary DNA deposited onto surfaces by contact, and data should be evaluated with caution, also considering the relatively low sample size. Indeed, the first transfer of DNA from saliva-moistened tips to the glass (T0) occurred within 1 h from the moistening, and different activities were performed by volunteers in this timeframe. This might explain the high variability noted in the DNA content recovered at T0.

In order to assess the effect of the alcohol-based hand sanitizer, a comparative analysis between the three different touch depositions (T0, T1, and T2) was performed. Results showed a reduction of human quantifiable DNA after the use of the sanitizer in both hands, without an increase of its degradation. However, the difference was not statistically significant (*p* > 0.05) in the samples of touch DNA recovered after deposition with the dominant hand. This might be due to the low amounts of DNA before the use of the hand sanitizer (median 19.8 pg/μl) and to the high inter-individual variability in DNA content deposited (IQ range, 6.4–50.3).

On the contrary, the use of the alcohol-based hand sanitizer led to a statistically significant reduction (T0 vs T1, with *p* < 0.05) of the DNA transferred to the glass surface from the non-dominant hand, where salivary DNA had been added. Nevertheless, it should be considered that the removal of DNA could be due both to the alcohol itself and to the rubbing action of the hands during hand sanitizing.

The difference was still significant after 1 h of normal activity (T0 vs T2, with *p* < 0.05), although this might be due to further transfer events occurring after the use of the hand sanitizer and is not with certainty associated with the effect of the sanitizer itself. The median DNA content decreased from T0 to T2, while an accumulation would likely result in an increase of the recovered transferred DNA amount. On this basis, the tendency to collect DNA on the hands from touched objects due to the stickiness of the sanitizer could not be observed, although further studies are required to confirm this result.

One hour after the use of the hand sanitizer (T2), the quantity of DNA recovered from samples containing skin-derived DNA and from those containing also salivary DNA did not statistically differ. The dramatically reduction of the yield of DNA is consistent with the mean percentage of saliva DNA loss of 81% in a single transfer event reported by Warshauer et al. [6].

This result suggests that, after the use of the hand sanitizer, touch DNA samples might fall below the recommended thresholds for DNA profiling. To evaluate the STR typing success and quality of genetic profiles obtained from touch DNA after the use of hand sanitizers, the reference profile obtained by buccal swab of the corresponding volunteer was compared to the generated STR profiles corresponding to the three depositions (T0, T1, T2).

Considering that all the 76 amplified samples provided at least 10 typed loci, even if the results were different between dominant and non-dominant hands, a detrimental effect of the hand sanitizer on DNA profiling was not observed. Accordingly, the number of profiles showing allele and locus dropout did not increase after the use of the hand sanitizer.

Samples deposited with the dominant hand at T0 mostly yielded mixed profiles without a major contributor and single source profiles, with 83.3% of informative profiles (matching to the donor with LR ≥ 10^6^). The only exceptions were two samples (D1F and D8F of the Table B, Supplementary data), which provided LR value of 10^−4^ and 10^3^, respectively. After hand sanitizing at T1, a decrease in the number of mixed profiles without a major contributor (from 50% to 9.1%) and an increase of mixed profiles with a major contributor (from 8.3 to 36.4%) matching the donor were seen, and all STR profiles were deemed informative to identify the donors (on the basis of the LR ≥ 10^6^ criteria). One hour after the use of the sanitizer, all profiles were still informative.

We hypothesize that at T0, within 1 h from handwashing and after various activities, both self- and non-self-DNA might be present on the hands. This is shown by the fact that informative profiles identifying the donor were 83.3%. The action of rubbing the hands might have led to the collection of self-DNA. This, becoming more prevalent than the non-self one, might explain the increase of informative profiles from 83.3% at T0 to 100% at T1. Nevertheless, it must be considered that only samples with > 15 pg/μl were selected for amplification, and the percentage of informative profiles could be lower when considering all samples.

Profiles generated from the non-dominant hand, to which salivary DNA had been added, were predominantly single source profiles at T0, but after the use of hand sanitizers, single source profiles dropped from 82.4 to 46.2% at T1, being replaced by mixed profiles with no major contributor (Fig. 3). This might also be connected to the rubbing activity between hands leading to a spread of salivary DNA and to the collection of non-self-DNA.

The results suggest that, if a perpetrator uses a hand sanitizer before committing a crime, the amount of DNA recovered from the touched surfaces will likely be reduced. However, if this does not fall below a certain threshold, an STR typing analysis would still be possible and could allow the identification of the perpetrator.

As a first limitation, despite a rigid protocol of fingerprint deposition, volunteers were free for 1 h before the first fingerprint deposition, and no activity recording sheet was provided. Also, the pressure applied to the glass surface could not be standardized. This could have contributed to the high inter-individual variability at T0, but the aim of our study was to evaluate the effect of the hand sanitizer, between T0 and T1, not the variables impacting the amount of DNA before T0.

Salivary DNA was transferred on the non-dominant hand, which is also generally less used, and the different use might influence the deposition of DNA [10].

However, activities only took place in the hour between T1 and T2, so that the bias connected to the use of the “less active” hand is only hypothesized for T2.

An analysis of the shedder status of the volunteers was not preliminary performed, due to the complexity of its assessment, which would require a dedicated study, with its own discussion and limits.

Another limitation of the study is represented by the relatively low sample size, although the number of tested samples is in line with past literature on touch DNA and was sufficient to provide significant data for our discussion.

As further limitation, the action of rubbing the hand might have also biased the evaluation of the effect of alcohol on the DNA rubbed between hands, by spreading self- or non-self-DNA and affecting the generated profiles. However, the chosen setup could better serve as a proxy for casework, since the CDC and WHO recommendations include the rubbing between hands.

As a final limitation, a rather precautionary threshold value for the DNA profiling was defined (set to 15 pg/μl), and this might have led to an overestimation of the percentages of informative profiles. Indeed, by lowering this threshold, a higher number of profiles not matching the donor with LR ≥ 10^6^ and more complex profiles would be expected. However, this threshold was use to better assess the effect of the hand sanitizer, reducing potential biases in DNA profiling evaluation related to the low amount of DNA.

## Conclusions

Touch DNA can be crucial in criminal cases to identify who may have made contact with surfaces, but the analysis of these samples remains challenging. Alcohol-based hand sanitizers are recommended since the beginning of the COVID-19 pandemic to prevent the virus transmission, and their effect on touch DNA analysis has been investigated in the present study. Our results have shown that the activity involving the use of an alcohol-based hand sanitizer reduced the amount of DNA deposited and recovered from touched surfaces, and the reduction is statistically significant when DNA-rich material (saliva) is deposited along with skin-derived DNA.

Nevertheless, when the amount of DNA does not fall below the recommended thresholds, the use of hand sanitizers does not reduce the STR typing success, and informative profiles matching to the reference sample can be obtained.

Future research could be developed by recruiting more volunteers, by depositing on different surfaces, by testing other formulations of hand sanitizers, or by evaluating multiple time points of deposition.

## Supplementary Information

Below is the link to the electronic supplementary material.

Supplementary file1 (PDF 360 KB)

## Data Availability

The datasets generated during and/or analyzed during the current study are available from the corresponding author on reasonable request.
