# Supplementary material for: Impact on touch DNA of an alcohol-based hand sanitizer used in COVID-19 prevention
Source: Int J Legal Med. 2023 Feb 24;137(3):645–53. doi: 10.1007/s00414-023-02979-2 (PMC9951825; doi:10.1007/s00414-023-02979-2)
Supplement: Supplementary file 1 — Supplementary file1 (PDF 360 KB) [file 414_2023_2979_MOESM1_ESM.pdf]

Impact on touch DNA of an alcohol-based hand sanitizer used for Covid-19 prevention

Content of the Supplementary

Table A. Summary of the quantification results giving the median and the interquartile range by brackets. All numbers are given in pg/µl. D: dominant hand; ND: non-dominant hand

Fig. A. Comparison of DNA content between F (female) and M (male) volunteers across T0 (before alcohol-based hand sanitizer), T1(immediately after the use of the alcohol-based hand sanitizer) and T2 (1 hour after the use of alcohol-based hand sanitizer)

Fig. B. Comparison of DNA content between the right (or dominant), touch DNA only, and left (non-dominant) to which salivary DNA was added, across T0 (before alcohol-based hand sanitizer), T1(immediately after the use of the alcohol-based hand sanitizer) and T2 (1 hour after the use of alcohol-based hand sanitizer)

Table B. Detailed genotyping results showing, for each sample, the profile outcome, whether single source profile, mixed profile with major contributor (MC) and mixed profiles with no MC. The maximum allele count (MAC), the minimum number of contributors (MNC) and the likelihood ratio (LR), expressing the matching to the donor profile are also shown. Dropout est.: probability of dropout as estimated by the software and (→) the corresponding LR value.

Table A. Summary of the quantification results giving the median and the interquartile range by brackets. All numbers are given in pg/µl. D: dominant hand; ND: non-dominant hand

	**T0 – before alcohol-based hand sanitizer**		**T1 - immediately after the use of the alcohol-based hand sanitizer**		**T2 - 1 hour after the use of alcohol-based hand sanitizer**	
	D	***ND***	***D***	***ND***	***D***	***ND***
Male	23.9 (10.4-60.0)	49.3 (22.4-174.8)	16.0 (8.8-33.4)	20.0 (8.8-38.8)	12.4 (6.4-31.4)	18.5 (10.5-59.4)
Female	19.8 (4.5-48.2)	148.7 (17.9-593.3)	5.3 (2.9-34.1)	46.7 (6.4-90.3)	12.7 (4.0-39.5)	16.5 (7.5-75.8)
All	19.8 (6.4-50.3)	77.5 (20.1-254.0)	14.0 (5.0-28.7)	26.3 (8.1-50.1)	12.7 (5.5-22.1)	16.5 (9.8-63.1)

Fig. A. Comparison of DNA content between F (female) and M (male) volunteers across T0 (before alcohol-based hand sanitizer), T1(immediately after the use of the alcohol-based hand sanitizer) and T2 (1 hour after the use of alcohol-based hand sanitizer)

Dominant hand	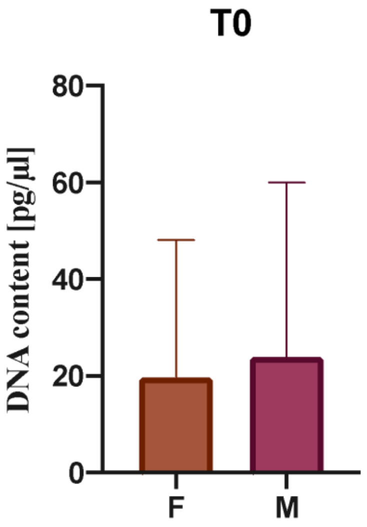<br>*p* = 0.59	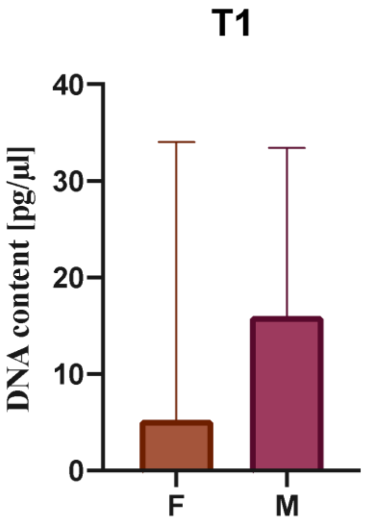<br>*p* = 0.38	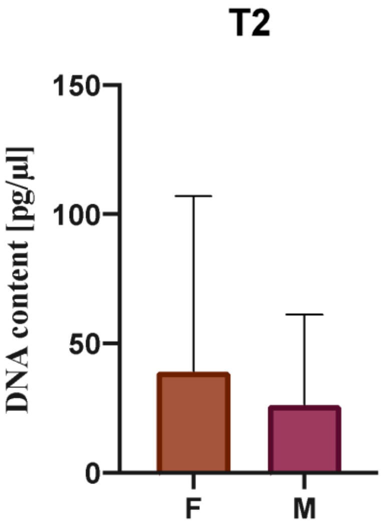<br>*p* = 0.89
Non-dominant hand	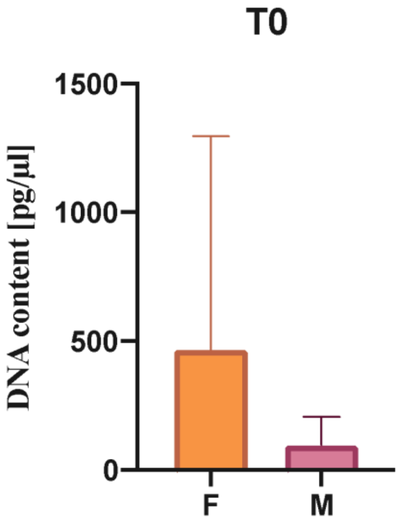<br>*p* = 0.35	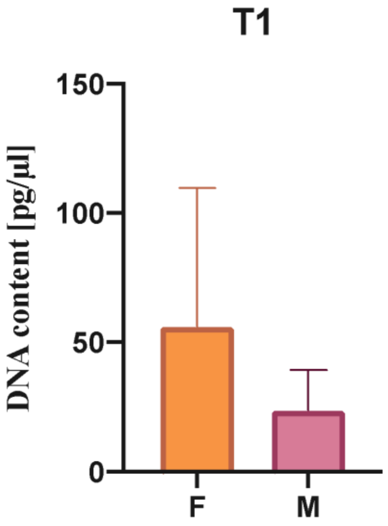<br>*p* = 0.25	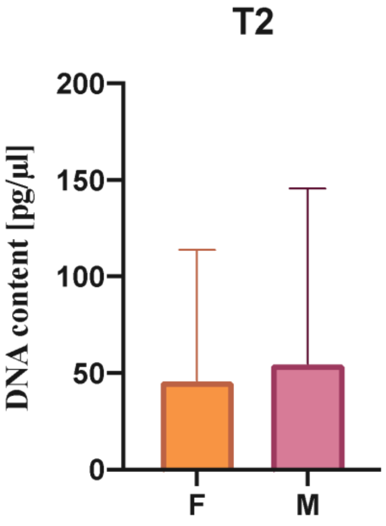<br>*p* = 0.78

Fig. B. Comparison of DNA content between the dominant and non-dominant hand, across T0 (before alcohol-based hand sanitizer), T1(immediately after the use of the alcohol-based hand sanitizer) and T2 (1 hour after the use of alcohol-based hand sanitizer). *=statistically significant

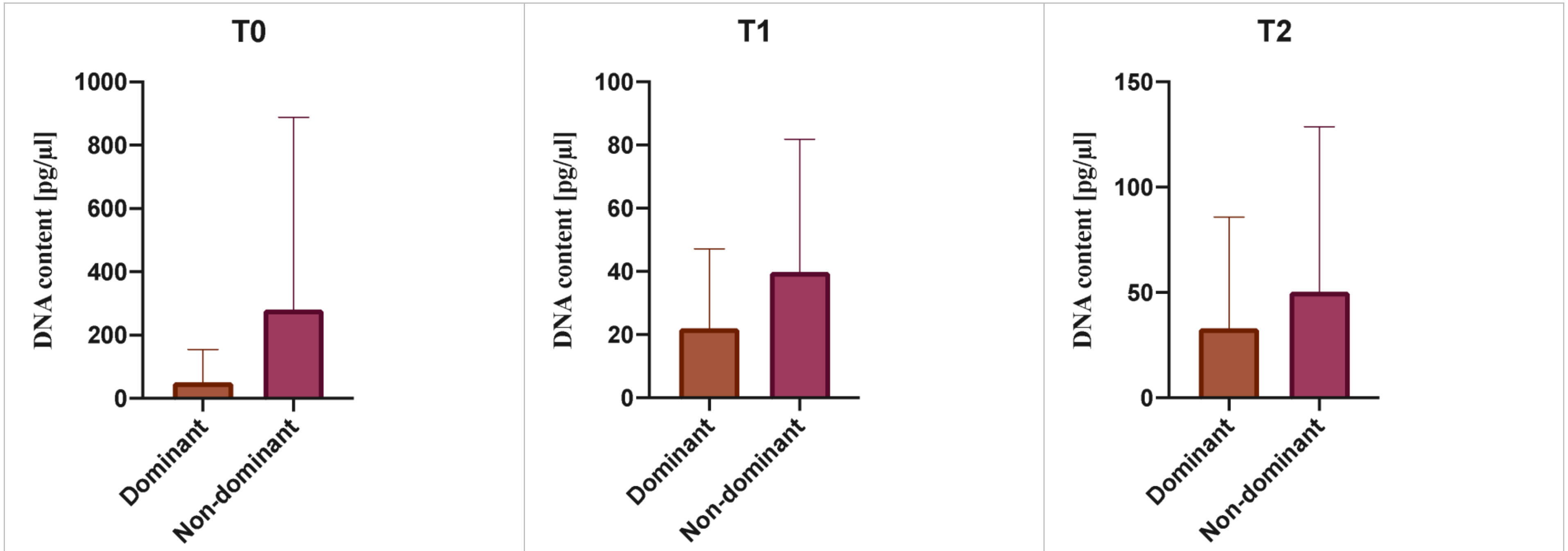

T0	T1	T2
$p = 0.02$*	$p = 0.02$*	$p = 0.10$

Table B. Detailed genotyping results showing, for each sample, the profile outcome, whether single source profile, mixed profile with major contributor (MC) and mixed profiles with no MC. The maximum allele count (MAC), the minimum number of contributors (MNC) and the likelihood ratio (LR), expressing the matching to the donor profile are also shown. Dropout est.: probability of dropout as estimated by the software and ($\rightarrow$) the corresponding LR value.

**Dominant hand**		**T0**	**T1**	**T2**
D1F	Profile	Mixed no MC	-	Mixed no MC
	MAC - MNC	3 - 2	-	4 - 2
	LR	3x10^{-4}	-	2x10^{14}
	Dropout est.	0.03 $\rightarrow$ 4.3x10^{-12}	-	0.04 $\rightarrow$ 1.9x10^{13}
D5F	Profile	Single source	Mixed no MC	Single source
	MAC - MNC	2 - 1	3 - 2	2 - 1
	LR	8.87x10^{25}	3x10^{14}	5.59x10^{28}
	Dropout est.	-	0.42$\rightarrow$ 6x10^{18}	-
D7 F	Profile	Single source	Single source	Single source
	MAC - MNC	2 - 1	2 - 1	2 - 1
	LR	2.48 x 10 ^{27}	8.3 x 10 ^{26}	1 x 10 ^{25}
	Dropout est.	-	-	-
D8F	Profile	Mixed no MC	Single source	Single source
	MAC - MNC	4 - 2	2 - 1	2 - 1
	LR	8x10^{1}	5.26x10^{32}	1 x10^{15}
	Dropout est.	0.53 $\rightarrow$4x10^{3}	-	0.24$\rightarrow$1.38 x10^{15}
D9F	Profile	Mixed no MC	Mixed with MC	-
	MAC - MNC	4 - 2	3 - 2	-
	LR	7x10^{12}	2.48x 10^{22}	-
	Dropout est	0.46$\rightarrow$6.92x10^{11}	0.3x10^{15}	--
D2M	Profile	Single source	Single source	Single source
	MAC - MNC	2 - 1	2 - 1	2 - 1
	LR	8x10 ^{24}	5x10 ^{28}	8x10 ^{28}
	Dropout est.	-	-	-
D4M	Profile	Single source	Single source	-
	MAC - MNC	2 - 1	2 - 1	-
	LR	2x10^{12}	7x10^{13}	-
	Dropout est	0.35 $\rightarrow$3x10^{12}	0.37$\rightarrow$1x10^{14}	
D5M	Profile	Mixed no MC	Single source	Single source
	MAC - MNC	4 - 2	2 - 1	2 - 1
	LR	2x10^{15}	2x10^{21}	9x 10^{22}
	Dropout est	0.21$\rightarrow$1.3x10^{13}	0.29$\rightarrow$3x10^{19}	0.16$\rightarrow$1.5x10^{19}
D6M	Profile	Mixed no MC	Mixed with MC	Single source
	MAC - MNC	3 - 2	4 - 2	2 – 1
	LR	2x10^{17}	2.4x10^{20}	1x 10^{2}
	Dropout est	0.23$\rightarrow$1x10^{16}	-	0.2$\rightarrow$5x10^{3}
D8M	Profile	Single source	Single source	-

	MAC - MNC	2 - 1	2 - 1	-
	LR	$1.2x10^{21}$	$1.5x10^{14}$	-
	Dropout est	$0.34 \rightarrow 3.1x10^{18}$	$4.1x10^{12}$	-
D13M	Profile	Mixed with MC	Mixed with MC	Mixed with MC
	MAC - MNC	3 - 2	3 - 2	4 - 2
	LR	$1.6x10^{15}$	$1.77x10^{19}$	$3x10^{14}$
	Dropout est.	$0.42 \rightarrow 4.8x10^{13}$	$0.34 \rightarrow 2.95x10^{17}$	$2.63x10^{14}$
D14M	Profile	Mixed no MC	Mixed with MC	Mixed no MC
	MAC - MNC	3 - 2	4 - 2	4 - 2
	LR	$3.7x10^{15}$	$1.22x10^{19}$	$2x10^{12}$
	Dropout est.	$0.34 \rightarrow 9.54x10^{13}$	-	-

**Non-dominant hand**		**T0**	**T1**	**T2**
ND2F	Profile	Single source	-	Single source
	MAC - MNC	2 - 1	-	2 - 1
	LR	$4x10^{16}$	-	$5x10^{20}$
	Dropout est.	$0.04 \rightarrow 5x10^{13}$	-	$0.04 \rightarrow 1x10^{19}$
ND3F	Profile	Single source	Single source	Single source
	MAC - MNC	2 - 1	2 - 1	2 - 1
	LR	$0.01 \rightarrow 1.3x10^{19}$	$5x10^{19}$	$4x10^{27}$
	Dropout est.	-	-	-
ND5F	Profile	Single source	Single source	Single source
	MAC - MNC	2 - 1	2 - 1	2 - 1
	LR	$8x\ 10^{25}$	$3.9x\ 10^{30}$	$1x10^{25}$
	Dropout est.	$0.35 \rightarrow 4x10^{17}$		
ND6F	Profile	Single source	-	Mixed with MC
	MAC - MNC	2 - 1	-	4 - 2
	LR	$4.76x10^{18}$	-	$2.88x10^{18}$
	Dropout est.	-	-	-
ND7F	Profile	Single source	Mixed no MC	Single source
	MAC - MNC	2 - 1	4 - 2	2 - 1
	LR	$4.5x10^{28}$	$1x10^{17}$	$4.6x10^{24}$
	Dropout est.	-	-	-
ND8F	Profile	Single source	Single source	-
	MAC - MNC	2 - 1	2 - 1	-
	LR	$5.25x10^{32}$	$1.95\ x\ 10^{30}$	-
	Dropout est.	-	-	-
ND9F	Profile	Single source	Single source	Mixed no MC
	MAC - MNC	2 - 1	2 - 1	3 - 2
	LR	$6.3x10^{27}$	$6.3x10^{27}$	$9\ x10^{9}$
	Dropout est	-	-	$0.62 \rightarrow 6x10^{8}$

ND11F	Profile	Single source	Mixed with MC	-
	MAC - MNC	2 - 1	3 - 2	-
	LR	2.4x 10^{20}	2.7x10^{18}	-
	Dropout est.	-	-	-
ND2M	Profile	Single source	Single source	Single source
	MAC - MNC	2 - 1	2 - 1	2 - 1
	LR	5x10^{28}	1.2x10^{29}	1.2x10^{29}
	Dropout est.	-	0.01→ 3.4x10^{27}	0.01→ 3.4x10^{27}
ND4M	Profile	Single source	Single source	Single source
	MAC - MNC	2 - 1	2 - 1	2 - 1
	LR	2x10^{15}	1x10^{16}	3.5x10^{10}
	Dropout est.	0.19 9x10^{15}	0.17 2x10^{16}	3.5x10^{10}
ND5M	Profile	Single source	Mixed no MC	Mixed no MC
	MAC - MNC	2 - 1	5 - 3	3 - 2
	LR	1x10^{20}	9x10^{18}	3x10^{14}
	Dropout est	0.19→1x10^{19}	0.21→7x10^{17}	0.1→3x10^{14}
ND6M	Profile	Mixed no MC	Mixed no MC	Mixed no MC
	MAC - MNC	4 - 2	4 - 2	3 - 2
	LR	3x10^{17}	5x10^{11}	3x10^{9}
	Dropout est	0.17→3x10^{17}	0.08→5x10^{11}	0.43→1.5 x10^{9}
ND7M	Profile	Single source	-	Single source
	MAC - MNC	2 - 1	-	2 - 1
	LR	2x10^{2}	-	3.4x10^{19}
	Dropout est	0.39→1.3x10^{5}	-	0.43→6x10^{11}
ND8M	Profile	Single source	-	Single source
	MAC - MNC	2 - 1	-	2 - 1
	LR	1x10^{18}	-	3.5x10^{19}
	Dropout est	0.2→1x10^{19}	-	0.34→1.5x10^{17}
ND11M	Profile	Single source	Mixed no MC	Single source
	MAC - MNC	2 - 1	4 - 2	2 - 1
	LR	1x10^{21}	3.5x10^{19}	5x10^{7}
	Dropout est	-	0.3→6x10^{17}	-
ND13M	Profile	Mixed with MC	Mixed no MC	-
	MAC - MNC	3 - 2	4 - 2	-
	LR	2.2x10^{21}	1.5x10^{19}	-
	Dropout est	-	0.3→5.5x10^{17}	-
ND14M	Profile	Mixed with MC	Mixed with MC	Mixed no MC
	MAC - MNC	4 - 2	4 - 2	4 - 2
	LR	1 x 10^{23}	7.56x 10^{19}	1x10^{18}
	Dropout est	-	0.3→6.58x10^{17}	0.32→3.4x10^{16}
